# The Role of Herpes Simplex Virus-1 Thymidine Kinase Alanine 168 in Substrate Specificity

**DOI:** 10.2174/1874091X00802010060

**Published:** 2008-05-09

**Authors:** Candice L Willmon, Django Sussman, Margaret E Black

**Affiliations:** 1 Department of Pharmaceutical Sciences, Washington State University, Pullman, WA; 2 Fred Hutchinson Cancer Research Center, Seattle, WA, USA

**Keywords:** Herpes Simplex Virus 1 Thymidine Kinase, ganciclovir, substrate specificity, gene therapy.

## Abstract

Herpes simplex virus type 1 (HSV) thymidine kinase (TK) has been widely used in suicide gene therapy for the treatment of cancer due to its broad substrate specificity and the inability of the endogenous human TK to phosphorylate guanosine analogs such as ganciclovir (GCV). The basis of suicide gene therapy is the introduction of a gene that encodes a prodrug-activating enzyme into tumor cells. After administration, the prodrug is selectively converted to a toxic drug by the suicide gene product thereby bringing about the eradication of the cancer cells. A major drawback to this therapy is the low activity the enzyme displays towards the prodrugs, requiring high prodrug doses that result in adverse side effects. Earlier studies revealed two HSV TK variants (SR39 and mutant 30) derived by random mutagenesis with enhanced activities towards GCV *in vitro* and *in vivo*. While these mutants contain multiple amino acid substitutions, molecular modeling suggests that substitutions at alanine 168 (A168) may be responsible for the observed increase in prodrug sensitivity. To evaluate this, site-directed mutagenesis was used to individually substitute A168 with phenylalanine or tyrosine to reflect the mutations found in SR39 and mutant 30, respectively. Additionally, kinetic parameters and the ability of these mutants to sensitize tumor cells to GCV in comparison to wild-type thymidine kinase were determined.

## INTRODUCTION

Thymidine kinase (TK), a key enzyme in nucleotide salvage metabolism, catalyzes the phosphorylation of thymidine (dT) to thymidylate (dTMP). While human TK is a strict thymidine kinase, the TK from Herpes Simplex Virus 1 (HSV) TK has a broad substrate specificity and can phosphorylate dT, dTMP, deoxycytidine, and various pyrimidine and guanosine analogs [1,2]. Guanosine analogs, such as the antiviral agents ganciclovir (GCV) and acyclovir (ACV), are selectively phosphorylated by HSV TK. Following initial phosphorylation of the analogs by HSV TK, endogenous enzymes are responsible for additional phosphorylation steps. In their triphosphate form, the guanosine analogs are incorporated into DNA and act as chain terminators to prevent further DNA synthesis [3-5].

The ability of HSV TK to phosphorylate nucleoside analogs is the basis of treatment for herpetic infections and for its use in gene therapy. Over the past 10-15 years HSV TK has been widely investigated for use in suicide gene therapy [6-8]. The principle of suicide gene therapy is to deliver a gene encoding a prodrug-activating enzyme to tumor cells. When the cells are subsequently treated with the non-toxic prodrug, the drug is converted into its toxic form only in the cells containing the suicide gene, thereby ablating the tumor cells. Although the HSV TK/GCV combination is currently being used in many clinical trials for a wide variety of cancers [9-4], inefficient gene delivery, poor enzyme activity

and GCV-related side effects restrict the therapeutic outcome of this approach.

**Fig. (1):**
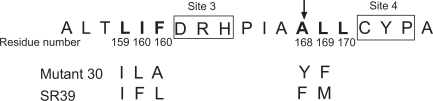
Deduced amino acid sequence of the two HSV TK mutants, mutant 30 and SR39. The top line shows the amino acid sequence of the residues 159-174 of wild-type HSV TK. Two highly conserved tripeptide motifs are boxed and denoted as Sites 3 and 4. The amino acids in bold in the top line denote codons that were targeted for the creation for the randomized libraries and subsequent mutations are shown in the following lines [15,16].

In order to improve the poor catalytic performance of the enzyme towards GCV, we employed protein engineering to optimize HSV TK for increased sensitivity to GCV. Our previous studies used random sequence mutagenesis to target six amino acid residues that neighbor two highly conserved tripeptide motifs shown to be involved in substrate binding (Fig. **1**) [15,16]. Several variants conferred enhanced prodrug sensitivity in rat C6 glioma transfected cells. In a xenograft tumor model, the most promising two mutants, mutant 30 and SR39, displayed impaired tumor growth at doses of GCV that did not impact wild-type HSV TK transfected tumors [17]. Molecular modeling of these multiple amino acid substituted variants led us to suggest that substitutions at position 168 may be crucial for the observed substrate alterations found in mutant 30 and SR39 [17]. To examine the role of A168 in substrate specificity we created individual substitutions to reflect mutations at A168 found in mutant 30 or SR39. These mutant and wild-type HSV TK enzymes were expressed and purified to near homogeneity from *E. coli*, and characterized for their kinetic properties. The substrate binding and catalytic efficiency information generated from theses studies were used to correlate functional enzyme data and modeled structures with three different substrates. Our results reveal that the phenylalanine (F) and tyrosine (Y) substitutions at the A168 are mainly accommodated by the side chain rearrangements that maintain interactions between HSV TK and the nucleoside analogs. Furthermore, these mutants were stably transfected in a rat C6 glioma cell line and evaluated for GCV sensitivity.

## MATERIALS AND METHODS

### Materials

The bacterial expression vector, pET23d, was purchased from Novagen (Madison, WI, USA). Oligonucleotides used forsite-directed mutagenesis and DNA sequencing were purchased fromOperon Technologies (San Pablo, CA). Restriction endonucleases used for screening mutants were purchased fromNew England Biolabs (Beverly, MA). Activated CH-Sepharose 4B and 3’-aminothymidine used to make the thymidine affinity column were purchased from Sigma (St. Louis, MO). Tritiated thymidine ([*methyl*-^3^H] thymidine, specific activity 90 Ci/mmol) was purchased from Amersham (Arlington Heights, IL) and [8-H]-ganciclovir (specific activities, 14.7 and 19.1 Ci/mmol) was purchased from Moravek Biochemicals (Brea, CA). All other reagents were purchased from Sigma (St. Louis, MO) unless otherwise denoted.

### Site Directed Mutagenesis

The oligonucleotide used to construct the A168F mutant is: 5’ GCAAGGAGGAAGGCGATGGGGTGGCG 3’. Oligonucleotides used to construct the A168Y mutant are: 5’ CTTCGACCGCCACCCCATCGCCTACCTCCTGTGC 3’ and 5’ GGAGGTAGGCGATGGGGTGGCGG 3’. The single-stranded DNA template for site-directed mutagenesis was isolated from pET23d:HSVTK (CJ236) [15]as described in Black and Hruby [18]. The single-stranded DNA was then used as a template for Kunkel-based site-directed mutagenesis[19,20] for the A168F mutant. The A168Y mutant was created using a Stratagene QuikChange kit (La Jolla, CA) according to the manufacturer’s directions, with dsDNA of pET23d:HSVTK as the backbone. Transformants were screened by restriction enzyme digestion for the loss of a *Bst*F51 site. Sequence analysis was performed on purified mutant plasmid DNA to confirm the mutation at the A168 position.

### Initial Evaluation of Thymidine and Prodrug Activity in *E. coli*

To evaluate the level of activity to thymidine (dT) and GCV, mutant and wild-type HSV TK plasmids were transformed into a thymidine kinase-deficient *E. coli* (BL21 (DE3) *tdk*^-^) and used to inoculate 3 mL of TK selection broth [21]. After an overnight incubation, cultures were serially diluted in 0.9% NaCl and spread onto 2 x YT containing carbenicillin (50 μg/mL)or TK selection plates containing different concentrations of prodrug as described in Black and Loeb [21]. Colony growth was scored after 16-24 hours at 37(C.

### Protein Expression and Purification

Expression and lysate preparation of wild-type and mutant HSV thymidine kinases were performed as described by Black *et al*. [15]. Enzymes were purified by affinity chromatography to near homogeneity using a 3’-aminothymidine sepharose column as described in Kokoris and Black [22]. Enzyme concentrations were determined using a Pierce BCA Protein Assay Kit (Rockford, IL) according to the manufacturer’s protocol, using BSA as the protein standard.

### Enzyme Assays and Kinetics

Thymidine kinase activity assays were performed using varying substrate and enzyme concentrations. The assay used to detect the phosphorylation of the nucleosides [*methyl*-^3^H]-thymidine and [8-3], except all washes were done at room temperature and the assays were performed at 37°C. The assays were conducted in triplicate three or more times. Data were plotted as the double reciprocal of velocity versus substrate concentration for each substrate. The Michaelis-Menten constant (K_m_) was determined using the double-reciprocal plots for each substrate. A conversion factor was determined for each [^3^H]-nucleoside monophosphate by measuring the cpms for a known number of moles of each tritiated nucleoside. The k_cat_ values were calculated using this conversion factor from the double reciprocal plots assuming one active site per monomer.

### Prodrug Sensitivity Assays

One µg of each DNA (pUB (vector alone), pUB:TK, pUB:TK A168F, pUB:TK A168Y, pUB:TK mutant 30, pUB:TK SR39) was used to transfect 1 x 10^5^ rat C6 glioma cells by lipofection using FuGENE 6 transfection reagent (Roche Diagnostics, Penzberg, Germany) at a 3:1 ratio according to the manufacturer’s directions. Immunoblots were performed to assess protein levels. Briefly, pools of transfectants were harvested and resuspended at 100,000 cells/μL in lysis buffer (for 2 mL: 2 μL 1 M DTT, 20 μL 1 M HEPES, 40 μL Nonidet P40 (Roche Diagnostics, Pernzberg, Germany) 2 μL MgAc_2_, H_2_O to final volume). The resuspended pellets were incubated on ice for 20 min and subjected to centrifugation at 4(C for 20 min to pellet debris. Samples (10 μL per well) were run on a 15% SDS gel, transferred to a nitrocellulose membrane and blocked with 3% gelatin in Tris-buffered saline. The membrane was probed with rabbit polyclonal TK antibody followed by goat anti-rabbit AP-conjugated antibody. The blot was developed using the AP Conjugate Substrate Kit (Bio-Rad, Hercules, CA). To determine the cytotoxicity of GCV pools of transfectants were transferred to 96-well microtiter plates at an initial density of 500 cells per well in DMEM plus supplements [17]. After cell adherence overnight, GCV (0-125 μM) was added in sets of eight wells for each concentration tested. The plates were incubated for 6 days at 37ºC in 5% CO₂₂_2_ at which time the redox-indicator dye Alamar Blue was added. Cell survival was determined several hours later as according to the manufacturer’s instructions and the data were plotted with a standard error of the mean bar. At least three replicates were performed.

### Structure Refinement and Molecular Modeling

Coordinates for the HSV TK structures with thymidine and GCV bound were obtained from the Protein Data Bank files 1KIM, 1KI2, and 2KI5 respectively [24,25]. Structures were refined using the Crystallography and NMR System (CNS) software version 1.1 [26]. Each of these structures was used to generate a model with either a phenylalanine or a tyrosine in position 168. All models (mutant and wild-type) were first subjected to 200 steps of gradient minimization, and then 1000 steps of torsion angle dynamics with slow-cooled annealing were performed from 1250 to 298 K with 25 K temperature drops per cycle. Molecular modeling and model analysis was performed using the computer graphics program XtalView [27]. Raster3D was used to generate figures [28].

## RESULTS

### Mutagenesis and Evaluation of Thymidine and Prodrug Sensitivity in*E. coli*

**To explore the role of the A168 substitutions seen in the HSV TK mutants, mutant 30[15] and SR39 [16] (Fig. **1**), site-directed mutagenesis of wild-type HSV TK was used to create two mutant HSV TK enzymes, A168F and A168Y. The A168 residue was substituted by a phenylalanine, as seen in SR39, or a tyrosine, as seen in mutant 30. An initial evaluation of the thymidine and prodrug activity of these mutants was performed in a thymidine kinase-deficient *E. coli*, BL21 (DE3) *tdk*^-^, as outlined in Materials and Methods (data not shown). Both A168 mutants were able to complement the TK-deficient *E. coli* under selective conditions. When GCV is used in a secondary selection, A168Y displays similar sensitivity as compared to wild-type HSV TK expressing cells. A168F, however, revealed an increased sensitivity to GCV as monitored by a reduced colony size relative to wild-type expressing cells.

### Enzyme Kinetics

Further biochemical characterization of the mutant and wild-type enzymes was done using dT and GCV as substrates. First, enzymes were purified by affinity chromatography to near homogeneity using a 3’-aminothymidine sepharose column [22]. Next, purified wild-type and mutant HSV TK enzymes were characterized using a filter binding assay previously described by Hruby and Ball [23]. The optimal substrate concentrations and enzyme amounts were determined for each substrate. All three enzymes (wild-type, A168F and A168Y) display non-linear Michaelis-Menten kinetics. The kinetic parameters were determined using double-reciprocal plots for each substrate.

### A168Y

As shown in Table **1**, the A168Y mutant displays a similar K_m_ value, or affinity, for dT compared to wild-type (K_m _= 1.06µM) with a value of 1.18 μM. However, the k_cat_₂, or turnover number, for this mutant is about 2% of the wild-type value with dT. Therefore, the overall efficiency (_₂_k_cat_/K_m_ = 0.70 s^-1^/μM) of this mutant is less than 2% wild-type (_₂_k_cat_/K_m_ = 38.02 s^-1^/μM). The A168Y mutant has a K_m_ value for GCV of 45.8 μM – equivalent to the wild-type K_m_ of 45.5 μM. Once again, this mutant displays a lower k_cat₂ _than wild-type, causing the efficiency value to be less than 11% that of wild-type. However, an important point to note is that endogenous thymidine within the cell competes with GCV for the active site. Therefore, the relative specificity constant for the prodrug and dT should be taken into consideration. For A168Y the relative specificity constant [(k_cat_/K_m (prodrug)_)/[(k_cat_/K_m (prodrug)_) + (k_cat_/K_m (dT)_)]] is over 5.3-fold higher than that for the wild-type enzyme with GCV as the substrate.

**Fig. (2):**
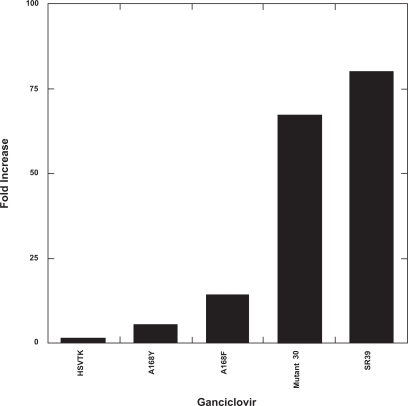
Mutant enzyme relative specificity differences compared to wild-type enzyme for ganciclovir. Using the equation, (k_cat_/K_m (GCV)_)/((k_cat_/K_m (GCV)_) + (k_cat_/K_m (dT)_)), the relative specificities were calculated. This equation takes into account the presence of endogenous thymidine that could compete with prodrug for the active site. The data are plotted as the fold increase from wild-type enzyme. Mutant 30 and SR39 values have been taken from Kokoris *et al.* [17] and [22], respectively.

Overall, the A168Y mutation in HSV TK causes a dramatic reduction in k_cat_ values for both dT and GCV whereas the K_m_ values remain unchanged. In comparison to kinetic values previously obtained for mutant 30 [17], the A168Y single mutation does not lead to as great an increase in activity towards GCV (Fig. **2**). Mutant 30 has a 67-fold increase in relative specificity for GCV, while the A168Y mutant has only a 5.3-fold increase in relative specificity for GCV.

### A168F

Both K_m_ (1.7µM) and k_cat_ (51.0 s^-1^) values for A168F with respect to thymidine are slightly higher than those of the wild-type enzyme (K_m_ = 1.06µM; k_cat_ = 40.3 s^-1^) (Table **1**). With GCV as the substrate, the A168F mutant has a K_m₂_ 5.3-fold lower than that of wild-type HSV TK and a k_cat_ of 91.1 s^-1^; 1.65-fold higher than that of the wild-type enzyme. Thus the catalytic efficiency (k_cat_/K_m_) of A168F for GCV is nearly 9-fold higher when compared to wild-type values. Although this mutant displays close to 9-fold greater preference for GCV than wild-type HSV TK based upon the relative specificity observed, A168F has a relative specificity constant 90% less than that of SR39 for GCV [22] (Fig. **2**).

### Prodrug Sensitivity Assays

**Fig. (3):**
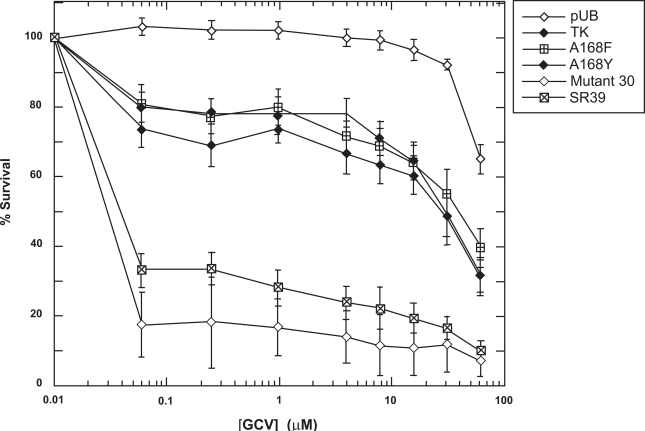
Sensitivity of TK-expressing rat C6 constructs to GCV. Pools of stable transfectants containing vector only (pUB), wild-type TK, A168F, A168Y, Mutant 30 and SR39 were constructed and transfected in rat C6 glioma cells and evaluated for prodrug sensitivity as described in Materials and Methods. After six days of prodrug treatment, the growth inhibition was determined by staining with Alamar Blue and fluorescence recorded at 530/590 nm. Each data point (mean ± SEM, n=3 performed with at least fifteen replicates) is expressed as a percentage of the value for control wells with no prodrug treatment.

To determine the prodrug activity of these mutants *in vitro*, mammalian expression vectors encoding the TK variants were constructed and used to transfect rat C6 glioma cells (see Materials and Methods). Immunoblot analyses of lysates from the pools of transfectants show similar expression levels for all of the mutants and wild-type TK, with no detectable expression in vector control pools (data not shown). Pools of stable transfectants were assayed for their level of GCV sensitivity over a drug range of 1-500 μM. Representative results of the prodrug sensitivities displayed by the TK mutants, wild-type TK and a vector control are shown in Fig. **3**. Little to no toxicity was observed with vector alone at the lower prodrug doses. Surprisingly, no increased sensitivity was observed with either A168F or A168Y in comparison to wild-type TK for GCV (Fig. **3**). Both mutant 30 and SR39 display about 100-fold increases in IC_50_ from wild-type TK (IC_50_ = 30 μM) and is similar what has been previously reported [15,16].

### Molecular Dynamics

**Fig. (4):**
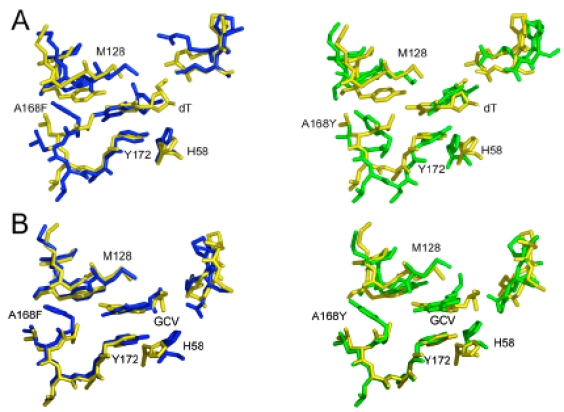
Superposition of HSV TK active site residues (residue numbers 58, 128-132,168-172, 222-225 (unlabeled in figure)). All three panels have consistent coloring: wild-type: yellow; A168F: blue; and A168Y: green. Models of wild-type and mutant proteins in complex with: A. Thymidine (thy); B. GCV.

Despite the replacement of a small hydrophobic side chain at position 168 with either an aromatic phenylalanine residue or bulky and polar tyrosine, many of the original contacts between protein and ligand are conserved after molecular dynamics/simulated annealing. In fact, these dramatic mutations are mainly accommodated by rearrangements in side-chain positions while the backbone undergoes relatively minor adjustments (Fig. **4**). These rearrangements allow the maintenance of hydrophobic and ionic interactions between the protein and both the nucleoside base analog and the sugar constituents of all ligands. Most notably, the hydrophobic tyrosine at position 172 (Y172) and methionine 128 (M128) residues sandwich the ring and define its placement within the binding site.

This conservation of the binding site is achieved largely because the substituted residues point directly away from the ligand and fit nicely into a hydrophobic pocket that is present in the wild-type structure, composed of residues leucine 169 (L169), F190, and L193. The filling of the binding pocket results in a slight rearrangement, presenting a highly complementary surface to GCV.

It should be noted that the final orientation of the mutated residue is somewhat dependent on its placement in the initial modeling. After molecular dynamics, one of two orientations is acquired; the residue either points toward the ligand (into contact with charged residues and disturbing the binding pocket) or away from it (as described above). These two structures converged to a similar total energy and might represent two structures that are present *in vivo. *However, because the binding pocket is greatly perturbed when the mutant residue points towards the ligand, a finding that is inconsistent with the kinetic data presented, we think it is highly unlikely the mutation results in this conformation.

## DISCUSSION

Although the use of HSV TK in suicide gene therapy for cancer has been widely investigated, the efficacy of this enzyme is rather limited due to low activity towards GCV and inefficient gene delivery to tumor cells. Additionally, GCV is myelosuppressive at doses that are required for complete tumor regression. While ACV is relatively nontoxic at high doses, it is not feasible for gene therapy due to the poor activity displayed towards it by HSV TK. In order to improve the efficacy of suicide gene therapy, protein engineering was used to optimize HSV TK for increased sensitivity to the prodrugs by Black *et al*. [15,16]. Earlier studies of two mutant enzymes show that mutant 30[15] and SR39 [16] confer increased GCV and ACV sensitivity to tumor cells. Molecular modeling of these multiple amino acid substituted mutants led us to hypothesize that the tyrosine or phenylalanine substitutions at the A168 position may cause neighboring side chains to move and thereby enlarge the active site allowing for greater prodrug accessibility to the active site and, in particular, lead to the increased prodrug sensitivity seen with these two variants [17]. In order to examine the role of the A168 substitutions seen in mutant 30 and SR39, site-directed mutagenesis was used to create individual A168 mutants and kinetic data were obtained.

The single A168Y mutation, corresponding to the substitution in mutant 30, led to the creation of an enzyme that has similar K_m_ valuescompared to wild-type values for both dT and GCV. A previous report by Mercer *et al*. [29] describing a site 4 HSV TK mutant Q30-3, which contains A168Y and L169F substitutions, concluded that the site 4 half site mutations are detrimental to substrate binding for both dT and GCV. In contrast, we found that the A168Y single substitution was active and, compared to wild-type HSV TK, has similar substrate specificity for thymidine and GCV. Other studies by Larder *et al*. [30] and Munir *et al*. [31] found that the A168 position when singly mutated with polar, uncharged amino acids maintained similar activity for dT compared to the wild-type HSV TK enzyme. Additionally, Larder *et al*. [30] found the A168T mutation displays not only a K_m_ value for dT similar to wild-type enzyme, but also for ACV. Recently, published work demonstrates that substitutions of A168 with aromatic side chains, such as F, S, or W, severely inhibits these enzymes from binding thymidine and GCV as substrate (with the exception of the F substitution for GCV) [32]. This same group also showed greatly reduced V_max_/K_m_ values for thymidine for the resulting mutants [32]. In our study the affinity towards the substrates was maintained by the A168Y mutant, however, the k_cat_ values for dT and GCVare substantially reduced relative to wild-type. Still, the relative specificity of this mutant HSV TK for GCV over dT is over 5-fold higher compared to wild-type HSV TK. However, our *in vitro* studies (Fig. **3**) suggest that a much greater relative specificity may be needed in order to achieve an advantage over wild-type enzyme to catalyze the phosphorylation of GCV in an environment consisting of other nucleosides, particularly thymidine.

Comparatively, mutant 30 has poorer kinetic parameters with dT than does the A168Y mutant [17]. The catalytic efficiency mutant 30 displays for dT is 0.03% wild-type, while A168Y has a value 1.84% that of wild-type. Mutant 30 preferentially utilizes GCV, as evidenced by the relative specificities and IC_50 _values that mutant 30 displays [17]. This is primarily as a result of the lower efficiency that mutant 30 has for the natural substrate and gives mutant 30 a 67-fold increase in relative specificity to GCV, respectively, compared to wild-type HSV TK. However, in comparison to the A168Y mutant, mutant 30 is clearly able to greater sensitize the rat C6 glioma cells to GCV. These results lead us to believe that A168 substitution in mutant 30 is important in improving its substrate specificity but more than likely it is not the only contributing factor to the enhancement of mutant 30-mediated GCV cytotoxicity.

Unlike A168Y, the A168F mutant displays major changes in prodrug substrate binding (GCV), but not dT binding. Furthermore, the turnover number of the A168F mutant was positively, albeit marginally, impacted; the observed k_cat_ of the A168F mutant is almost double that of wild-type. Compared to wild-type HSV TK, the relative specificity A168F displays towards GCV is 9-fold higher, while the relative specificity SR39 displays is 85-fold higher for GCV. Like mutant 30, SR39 displays poor kinetics for dT, with a catalytic efficiency only 0.53% wild-type for dT [22], while A168F has a catalytic efficiency about 79% wild-type HSV TK. The low efficiency of SR39 towards dT presumably results in less competition at the active site between dT and GCV than would be seen with the A168F mutant and is likely to be responsible for the increased relative specificity kinetically and in the tumor cell killing that SR39 displays towards GCV compared to A168F.

## CONCLUSIONS

The two single amino acid substitutions at A168 appear not to be singularly responsible for the increased sensitivity towards GCV seen with SR39 or mutant 30 *in vitro *or *in vivo*. Indeed kinetic changes observed provide some insights to the impact these substitutions have. For A168Y the K_m_ for the substrates is unchanged from the wild-type enzyme, whereas the turnover number (k_cat_) is impaired most dramatically for dT (2% wild-type HSV TK). In contrast, while the A168F K_m_ value for dT is similar to wild-type HSV TK, significant improvements in GCV binding and k_cat _have occurred. Molecular modeling suggests that an enlargement of the active site induced by the introduction of large aromatic residues may be partially responsible for the improved affinity of the HSV TK mutants for GCV. The additional mutations in the previously characterized HSV TK variants (mutant 30 and SR39) might serve to change the shape of the binding pocket even further. Although the A168F and A168Y mutants display improved relative specificities for GCV, the *in vitro *cell culture studies demonstrate that these substitutions do not provide significant enough improvement to alter the sensitivity of tumor cells expressing these mutants. Therefore, we believe other substitutions found within the active pocket, in conjunction with the F and Y substitutions studied here, are important contributors to the dramatic enhancement of mutant 30 and SR39 in the tumor cell killing and xenograft studies previously demonstrated [15-17, 22].

## Figures and Tables

**Table 1.** Kinetic Parameters of Mutant and Wild-Type Thymidine Kinases

** **	**HSV TK**	**A168Y**	**A168F**
**Thymidine**			
K_m_ (μM)	1.06*^a^*	1.18	1.70
k_cat_ (s^-1^)	40.30	0.83	51.00
k_cat_/K_m_ (s^-1^/μM)	38.02	0.70	30.00
**Ganciclovir**			
K_m_ (μM)	45.50	45.80	8.60
k_cat_ (s^-1^)	55.30	6.00	91.10
k_cat_/K_m_ (s^-1^/μM)	1.22	0.13	10.59
Relative Specificity*^b^*	0.03	0.16	0.26

*^a^*Values are the averages of at least three independent measurements. Standard error of the means are within a range of ± 20%. *^b^*Relative Specificity = (k_cat_/K_m (GCV)_)/((k_cat_/K_m (GCV)_) + (k_cat_/K_m (dT)_)).
